# Red Nucleus Interleukin-6 Evokes Tactile Allodynia in Male Rats Through Modulating Spinal Pro-inflammatory and Anti-inflammatory Cytokines

**DOI:** 10.3389/fnmol.2022.820664

**Published:** 2022-04-08

**Authors:** Qing-Qing Yang, Hao-Nan Li, Yu-Tong Xia, Xue Tian, Fan Feng, Jian Yang, Ya-Li Xu, Juan Guo, Xiao-Qi Li, Jun-Yang Wang, Xiao-Yan Zeng

**Affiliations:** ^1^Department of Laboratory Medicine, The First Affiliated Hospital of Xi’an Jiaotong University, Xi’an, China; ^2^Department of Pathogenic Biology and Immunology, Xi’an Jiaotong University Health Science Center, Xi’an, China

**Keywords:** allodynia, interleukin-1β, interleukin-6, interleukin-10, red nucleus, spinal cord, transforming growth factor-β, tumor necrosis factor-α

## Abstract

Our previous studies have clarified that red nucleus (RN) interleukin (IL)-6 is involved in the maintenance of neuropathic pain and produces a facilitatory effect by activating JAK2/STAT3 and ERK pathways. In this study, we further explored the immune molecular mechanisms of rubral IL-6-mediated descending facilitation at the spinal cord level. IL-6-evoked tactile allodynia was established by injecting recombinant IL-6 into the unilateral RN of naive male rats. Following intrarubral administration of IL-6, obvious tactile allodynia was evoked in the contralateral hindpaw of rats. Meanwhile, the expressions of pro-inflammatory cytokines tumor necrosis factor-α (TNF-α), IL-1β, and IL-6 were elevated in the contralateral spinal dorsal horn (L4–L6), blocking spinal TNF-α, IL-1β, or IL-6 with neutralizing antibodies relieved IL-6-evoked tactile allodynia. Conversely, the levels of anti-inflammatory cytokines transforming growth factor-β (TGF-β) and IL-10 were reduced in the contralateral spinal dorsal horn (L4–L6), an intrathecal supplement of exogenous TGF-β, or IL-10 attenuated IL-6-evoked tactile allodynia. Further studies demonstrated that intrarubral pretreatment with JAK2/STAT3 inhibitor AG490 suppressed the elevations of spinal TNF-α, IL-1β, and IL-6 and promoted the expressions of TGF-β and IL-10 in IL-6-evoked tactile allodynia rats. However, intrarubral pretreatment with ERK inhibitor PD98059 only restrained the increase in spinal TNF-α and enhanced the expression of spinal IL-10. These findings imply that rubral IL-6 plays descending facilitation and produces algesic effect through upregulating the expressions of spinal pro-inflammatory cytokines TNF-α, IL-1β, and IL-6 and downregulating the expressions of spinal anti-inflammatory cytokines TGF-β and IL-10 by activating JAK2/STAT3 and/or ERK pathways, which provides potential therapeutic targets for the treatment of pathological pain.

## Introduction

Neuropathic pain is a refractory disorder following injury or disease of the somatic sensory nervous system (Jensen et al., 2011), characterized by the presence of spontaneous pain, allodynia, and hyperalgesia. The development of neuropathic pain requires a large number of different mechanisms, and the peripheral and central sensitization induced by overproduction of neurotransmitters, neuropeptides, and enzymes are considered to be the basis of pathological pain generation and maintenance (Cohen and Mao, 2014). Additionally, a growing number of studies display that immune molecules also engage in the sensitization of the nerve system and the development of chronic pathological pain. For example, locally elevated pro-inflammatory cytokines such as interleukin-1 (IL-1β), IL-6, and tumor necrosis factor-α (TNF-α) and decreased anti-inflammatory cytokines such as IL-10 can boost peripheral neural activity (Okamoto et al., 2001; Ozaktay et al., 2002). Increased IL-1β, IL-6, and TNF-α are also detected in the spinal cord and some pain-associated supraspinal regions, including the cerebral cortex, midbrain periaqueductal gray, hippocampus, amygdala, and rostral ventromedial medulla (RVM), blocking the activity of IL-1β, IL-6, or TNF-α attenuates pathological pain (Winkelstein et al., 2001; Milligan et al., 2003; Jia et al., 2007; Wei et al., 2008; Al-Amin et al., 2011; Gui et al., 2016; Ho et al., 2020; Guo et al., 2021).

The red nucleus (RN) is a distinct supraspinal zone of the ventral midbrain, usually divided into magnocellular (mRN) and parvocellular (pRN) parts (Paxinos and Watson, 2007). In primates, the pRN mainly connects with the inferior olivary nuclei, dentate nucleus, and cerebellum, and the mRN is the cradle of the rubrospinal tract (RST). The RST neurons are widely spread in laminae IV, V, and VI and the dorsal portion of laminae VII of the spinal cord (Küchler et al., 2002). Besides regulation in locomotion, such as posture maintenance, conditioning reflex, learning of acquired motor behavior, and detailed reach-to-grasp of hand (Whishaw et al., 1992; Zelenin et al., 2010; Yajima et al., 2012; Rizzi et al., 2019), the RN also acts in sensory modulation. Functional magnetic resonance imaging (fMRI) study directly proves that the RN is about pain processing (Bingel et al., 2002), and peripheral noxious stimuli alter the electrophysiological activities of rubral neurons (Matsumoto and Walker, 1991; Steffens et al., 2000). Continuous migraine is accompanied by the activation of the RN region (Matharu and Goadsby, 2005), and migraine without aura has interrupted RN-substantia nigra functional connection (Huang et al., 2019). We recently demonstrated the RN regulates spared nerve injury (SNI)-induced neuropathic pain *via* pro-inflammatory cytokines IL-1β, TNF-α, and IL-33 and anti-inflammatory cytokines IL-10 and transforming growth factor-β (TGF-β), in which pro-inflammatory cytokines play an algesic effect and anti-inflammatory cytokines play analgesic effect, suggesting that rubral cytokines play vital roles in the modulation of neuropathic pain (Li et al., 2008, 2021; Wang et al., 2008, 2012, 2015).

Interleukin-6 is a versatile cytokine associated with multiple biological processes (Hunter and Jones, 2015). Accumulated evidence shows that IL-6 has a hand in pain regulation by involving nociceptive plasticity and sensitization (Melemedjian et al., 2014). In the painful pathological state, the increased expression of IL-6 occurs in the peripheral injured nerve as well as central nervous system (Okamoto et al., 2001; Dubový et al., 2010; Wei et al., 2013). The ectopic impulse of allodynia generated by trigeminal ganglion neurons is mediated by IL-6 after infraorbital nerve injury (Liu et al., 2021). *In vivo* injection of IL-6 evokes pain hypersensitivity in normal animals and aggravates the pain-associated behaviors in rats with nerve lesions (Oka et al., 1995; DeLeo et al., 1996; Vissers et al., 2005; Brenn et al., 2007). Our recent study has shown that IL-6 is constitutively expressed in the RN of naive rats and upregulated at 3 weeks post peripheral nerve injury. Blocking rubral IL-6 with neutralizing antibodies alleviates neuropathic pain, while intrarubral administration of exogenous IL-6 can induce tactile allodynia in normal rats (Ding et al., 2016), implying rubral IL-6 mediates the maintenance of neuropathic pain and produces a facilitatory effect. Further studies show that rubral IL-6 activates the janus kinase 2/signal transducer and activator of transcription 3 (JAK2/STAT3) and extracellular signal-regulated kinase (ERK) pathways, thus promoting the production of local TNF-α and IL-1β and regulating the maintenance of chronic pain (Ding et al., 2018; Yang et al., 2020). In this study, we further proved that rubral IL-6 plays descending facilitation and produces an algesic effect through upregulating the expressions of spinal pro-inflammatory cytokines TNF-α, IL-1β, and IL-6 and downregulating the expressions of spinal anti-inflammatory cytokines TGF-β and IL-10 by activating JAK2/STAT3 and/or ERK pathways.

## Materials and Methods

### Animals

Male adult Sprague-Dawley rats (200–250 g) were supplied by the Experimental Animal Center (Shaanxi, China). The rats were reared in an air-conditioned environment (23 ± 1°C) with a 12/12 h diurnal cycle, feeding enough chow and water. For our animal experiments, we followed the guidelines of the institutional Biomedical Ethics Committee of Xi’an Jiaotong University and the International Association for the Study of Pain, and all efforts were made to minimize the number of animals used and their suffering.

### Intracerebral Intubation and Drug Injection

The rat was intraperitoneally (i.p.) anesthetized with urethane (1.4 g/kg) and immobilized in a stereotaxic apparatus. After sterilization of skin, the cranium was exposed, and a hole was drilled above the RN. A metal cannula with a stainless plug was then inserted vertically 2 mm above the left RN according to the following stereotaxic coordinates: 5.2–6.7 mm behind the bregma, 0.6–1.4 mm beside the midline, and 6.4–7.4 mm under the cortex (Paxinos and Watson, 2007). Then the implant was fixed with dental cement, and the rats were placed in a warm environment for recovery. To avoid infection, penicillin was injected i.p. at 0.2 million units per day for a continuous 3 days post operation.

**FIGURE 1:**
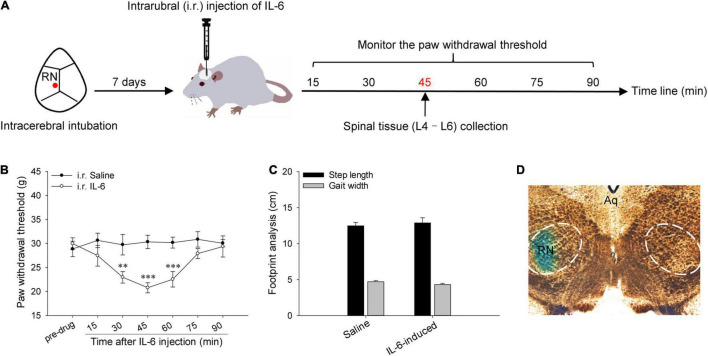
Red nucleus IL-6-evoked tactile allodynia. **(A)** Experimental schemes for studying the expressions of spinal pro-inflammatory and anti-inflammatory cytokines in rubral IL-6-evoked tactile allodynia rats. **(B)** Intrarubral injection of 20 ng IL-6 evoked significant tactile allodynia in the contralateral hindpaw of naive rats compared with normal saline (*n* = 7 rats/group). **(C)** Footprint analysis indicated that intrarubral injection of 20 ng IL-6 did not affect the locomotion of naive rats (*n* = 6 rats/group). **(D)** The injection site was verified to be within the RN by 2% direct blue 1. **P < 0.01 and ***P < 0.001. RN, red nucleus; Aq, aqueduct of midbrain.

A week after intubation, the plug was removed from the cannula under isoflurane inhalation anesthesia (RWD Life Science Co., Shenzhen, China), and a 1.0 μl syringe 2 mm beyond the cannula was used for intrarubral injection of drugs (0.5 μl). The following drugs were used: recombinant rat IL-6 (Sigma, United States, 40 ng/μl), JAK2 suppressant AG490 (Abcam, United Kingdom, 10 μg/μl), and ERK suppressant PD98059 (Abcam, United Kingdom, 5.0 μg/μl). The IL-6 was prepared using normal saline. The suppressants were first melted with dimethyl sulfoxide (DMSO) and then diluted with normal saline to maintain a DMSO with a final concentration of 10% (v/v). The concentration of drugs used in this study was the same as used in previous studies and was effective (Ding et al., 2016, 2018). The injection site was confirmed using 2% direct blue 1 at the end of the experiment (Figure 1D).

### Induction of Interleukin-6-Evoked Tactile Allodynia

A week after intubation, a single dosage of recombinant rat IL-6 (20 ng in 0.5 μl) was delivered to the left RN of naive rats to evoke tactile allodynia according to our previous study (Ding et al., 2016). Equal amounts of IL-6 pre-neutralized with anti-IL-6-Ab (500 ng, Abcam, United Kingdom) and normal saline were injected as negative controls. When the rubral IL-6 function was at its peak point (45 min post injection), the rats were sacrificed under deep anesthesia, and L4-L6 spinal cord tissues were collected (Figure 1A).

### Intrathecal Injection of Drugs

**FIGURE 2:**
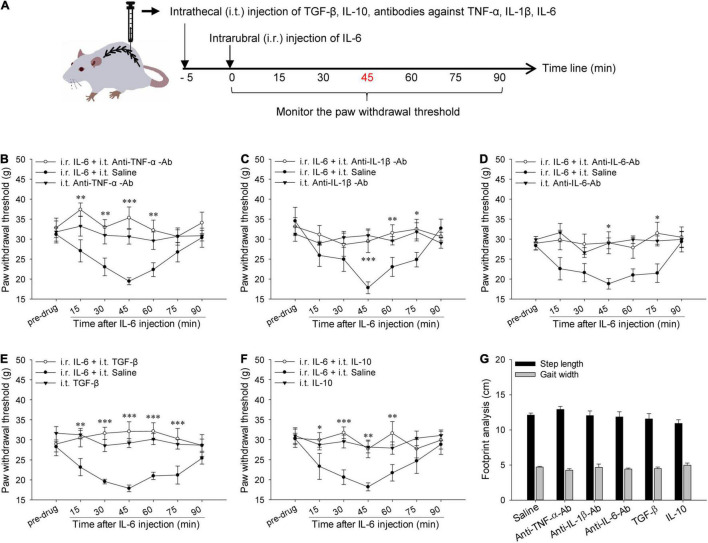
Actions of spinal pro-inflammatory and anti-inflammatory cytokines in rubral IL-6-evoked tactile allodynia. **(A)** Experimental schemes for studying the actions of spinal pro-inflammatory and anti-inflammatory cytokines in rubral IL-6-evoked tactile allodynia. **(B–D)** Intrathecal pre-injection of 2.0 μg anti-TNF-α-Ab, 1.0 μg anti-IL-1β-Ab, or 1.0 μg anti-IL-6-Ab alleviated rubral IL-6-evoked tactile allodynia compared to the normal saline (*n* = 7 rats/group). **(E,F)** Intrathecal pre-injection of 10 ng TGF-β or 300 ng IL-10 lessened rubral IL-6-evoked tactile allodynia compared with normal saline control (*n* = 7 rats/group). **(G)** Intrathecal injection of anti-TNF-α-Ab, anti-IL-1β-Ab, anti-IL-6-Ab, recombinant TGF-β, or IL-10 did not alter the locomotion of naive rats (*n* = 6 rats/group). *P < 0.05, **P < 0.01 and ***P < 0.001.

As illustrated in Figure 2A, the drug was intrathecally injected 5 min before intrarubral injection of IL-6 to explore the roles of spinal inflammatory cytokines. The rat was anesthetized by inhalation of isoflurane, and a 50 ml centrifuge tube was put under the rat’s abdomen so that its lumbar spine was flexed at the level of the L4–L5 vertebrae. Then a 30-gauge needle attached to a 10 μl Hamilton syringe was inserted at an angle of approximately 20° into the tissue between the L4 and L5 spinous processes. The needle was then advanced to the notch between the spinous process and the transverse process and was pierced into the vertebral space at an angle of approximately 10°. The presence of the tail-flick reflex signifies successful intrathecal injection (Li et al., 2020). Drugs (10 μl) used in intrathecal injection include anti-TNF-α-Ab (Proteintech, Wuhan, China, 0.2 μg/μl), anti-IL-1β-Ab (Abcam, United Kingdom, 0.1 μg/μl), anti-IL-6-Ab (Abcam, United Kingdom, 0.1 μg/μl), recombinant rat IL-10 protein (Cloud-Clone Corp., Wuhan, China, 30 ng/μl), and recombinant rat TGF-β1 protein (Cloud-Clone Corp., Wuhan, China, 1.0 ng/μl). All of these drugs were prepared using normal saline, and the doses were considered effective in reported pain studies (Souter et al., 2000; Chacur et al., 2009; Chen et al., 2013; Wu et al., 2018).

### Mechanical Pain Threshold Test

To assess the response of rats to tactile stimulus, the tactile paw withdrawal threshold (PWT) was determined using a Dynamic Plantar Aesthesiometer (Ugo Basile, Italy). The test was performed by an experimenter blinded to the drug intervention according to the procedures described by the manufacturer and as previously reported (Liang et al., 2018; Li et al., 2021). Before the formal test, the rat was placed in a transparent organic plastic box with a grid-like metal plate at the bottom for 20 min. Then, the start key on the microprocessor was pressed, and a blunt wire (0.5 mm in diameter) was lifted to vertically stimulate the lateral of the rat hindpaw with increasing intensity ranging from 0 to 50 g within 30 s. Only when the rat quickly flinched did the hindpaw indicate a positive withdrawal reaction, and the PWT value was automatically recorded.

### Footprint Test

To test whether the intrarubral and intrathecal injection of drugs affect the motor function of rats, footprint tests were conducted separately on the second day after mechanical pain threshold test at the peak effect time point after reinjection of the drugs (45 min post intrarubral injection of IL-6; 50 min post intrathecal injection of TGF-β, IL-10, or antibodies against TNF-α, IL-1β, and IL-6). The hindpaws of the rat were sprayed with non-toxic black dye, and the rat was then allowed to proceed normally along a transparent plastic channel (70 × 12 × 15 cm^3^, length × width × height) laying with a layer of white paper. After the end, 5–6 steps of footprints were selected to measure the step length and gait width (Li et al., 2021).

### Quantitative Real-Time Polymerase Chain Reaction

Total RNA was extracted from spinal tissues using the Trizol reagent (Beyotime, Shanghai, China), and RNA (1.0 μg) was DNase-treated and reverse-transcribed using the reverse transcriptase 2 first strand kit (Beyotime, Shanghai, China) depending on the manufacturer’s recommendation. The spinal cord tissues from two rats were mixed into a single sample, and Quantitative Real-Time Polymerase Chain Reaction (qRT-PCRs) were performed in technical triplicates for each primer set. qRT-PCR was run using an ABI instrument (MX3005P) real-time PCR system, and the reaction system contained 10 μl 2 × SYBR Mix (ABclonal, Wuhan, China), 2 μl cDNA, 0.4 μl sense and antisense primer, and 7.2 μl nuclease-free water to make a total volume of 20 μl. The sequences of primers (Sango Biotech, Shanghai, China) are listed in Table 1. The amplification reaction was performed within the linear range of the logarithmic phase unless otherwise specified. Thermal cycling conditions included pre-degeneration at 95°C for 3 min, 40 PCR cycles of denaturation at 95°C for 5 s, and extension at 60°C for 30 s. Quantitative real-time PCR results were calculated using the 2^–ΔΔCt^ method.

**TABLE 1:** Primer sequences details.

Gene	Primer sequences (sense/antisense) (5′→3′)	Size (bp)	Tm (°C)	GenBank™ accession number
TNF-α	ATGGGCTCCCTCTCATCAGTTCC	114	64	NM_012675
	GCTCCTCCGCTTGGTGGTTTG			
IL-1β	CTCACAGCAGCATCTCGACAAGAG	95	64	NM_031512
	TCCACGGGCAAGACATAGGTAGC			
IL-6	ACTTCCAGCCAGTTGCCTTCTTG	110	64	NM_012589
	TGGTCTGTTGTGGGTGGTATCCTC			
TGF-β	GACCGCAACAACGCAATCTATGAC	94	63	NM_021578
	CTGGCACTGCTTCCCGAATGTC			
IL-10	CTGCTCTTACTGGCTGGAGTGAAG	89	63	NM_012854
	TGGGTCTGGCTGACTGGGAAG			
β-actin	CTGAGAGGGAAATCGTGCGTGAC	93	64	NM_031144
	AGGAAGAGGATGCGGCAGTGG			

### Western Blotting

The total proteins of spinal cord were extracted with refrigerant radioimmunoprecipitation assay (HEART, Xi’an, China) containing protease inhibitors and phosphatase inhibitors (Bimake, United States) in assist of a homogeneous instrument. Then a BCA protein assay kit (Dingguo, Beijing, China) was utilized to quantify the concentration, and 20 μg/lane protein was loaded onto and separated in 10–15% (w/v) sodium dodecyl sulfate-polyacrylamide gel. The target proteins were transferred from the gel to a 0.45 μm polyvinylidene difluoride membrane. Following a 2 h incubation in 5% milk, the membranes were reacted with the primary antibodies overnight at 4°C. The primary antibodies used were as follows: anti-TNF-α-Ab (Proteintech, Wuhan, China, 1:500), anti-IL-1β-Ab (Wanleibio, Shenyang, China, 1:500), anti-IL-6-Ab (Proteintech, Wuhan, China, 1:1,000), anti-TGF-β1-Ab (Abcam, United Kingdom, 1:500), anti-IL-10-Ab (Proteintech, Wuhan, China, 1:1,000), and anti-GAPDH-Ab (Proteintech, Wuhan, China, 1:5,000). Then, the membranes were reacted with the secondary antibody horseradish peroxidase (HRP)-conjugated anti-rabbit IgG (Zsbio, Beijing, China, 1:5,000) for 1 h. Finally, the membranes were developed using an ECL substrate. Each target protein and its corresponding internal reference GAPDH were separately detected using the same membrane. The chemiluminescent images were captured using the Fusion FX5 camera system, and semi-quantitative analysis was conducted using the Image J software (National Institutes of Health, Bethesda, MA, United States). The value was normalized by GAPDH.

### Immunohistochemistry

Under deep anesthesia, the rats were instilled with 200 ml of normal saline and then 300 ml of 4% paraformaldehyde through the aorta of the heart. The caudal intumescentia lumbalis part (L4–L6) of the rat spinal cord was dissected with the final tips as marks. The spinal tissues were postfixed overnight, dehydrated in turn to 20 and 30% sucrose solution, and then were sectioned in a cryostat at a 10 μm thickness. One section from every 100 μm was selected, and 3–4 sections per animal were used for staining. After antigen retrieval and goat serum blocking, the slices were reacted overnight at 4°C in anti-TNF-α-Ab (Abcam, United Kingdom, 1:300), anti-IL-1β-Ab (Abcam, United Kingdom, 1:300), anti-IL-6-Ab (Proteintech, Wuhan, China, 1:300), anti-TGF-β1-Ab (Abcam, United Kingdom, 1:300), and anti-IL-10-Ab (Proteintech, Wuhan, China, 1:300). As negative controls, primary antibodies were either abandoned or substituted with normal rabbit IgG. Then, the tissue slices were reacted with HRP-conjugated goat anti-rabbit IgG (BosterBio, Wuhan, China) at 37°C for 30 min and stained with DAB. The Axio Scope A1 microscope (Carl Zeiss, Germany) was used to capture the histological photos. The Image J software was used to analyze the mean optical density (MOD) of the spinal dorsal horn (laminae I–VI).

### Statistical Analysis

All data were expressed as mean ± standard error. One-way analysis of variance (ANOVA) with the Holm-Sidak *post-hoc* test was utilized to measure the differences in mRNA and protein expression in various groups. Two-way repeated measures of ANOVA with the Holm-Sidak *post-hoc* test were utilized to measure the differences in PWT in various groups. *P* < 0.05 was considered statistically significant.

## Results

### Interleukin-6-Evoked Tactile Allodynia

A week post intrarubral intubation, a single dose of 20 ng recombinant rat IL-6 was administered into the left RN to evoke tactile allodynia (Figure 1A). As depicted in Figure 1B, intrarubral injection of IL-6 dramatically decreased the PWT of the contralateral hindpaw and showed temporary but marked tactile allodynia compared with normal saline (*F* = 3.759, *P* < 0.01), and the peak effect arose at 45 min post injection (20.79 ± 1.057 g, *t* = 4.401, *P* < 0.001). Nevertheless, intrarubral injection of IL-6 did not change the PWT of the ipsilateral hindpaw compared with normal saline (*F* = 1.481, *P* > 0.05), and the PWT was 27.04 ± 1.267 g at 45 min post injection. Intrarubral injection of IL-6 pre-neutralized with anti-IL-6-Ab (data were not shown) or normal saline had no obvious effects on the PWT of rats. Additionally, intrarubral injection of IL-6 did not affect the step length and gait width of rats, which meant there was no effect on the locomotion of rats (Figure 1C).

### Intrarubral Injection of Interleukin-6 Induces the Expressions of Spinal Pro-inflammatory Cytokines Tumor Necrosis Factor-α, Interleukin-1β, and Interleukin-6

**FIGURE 3:**
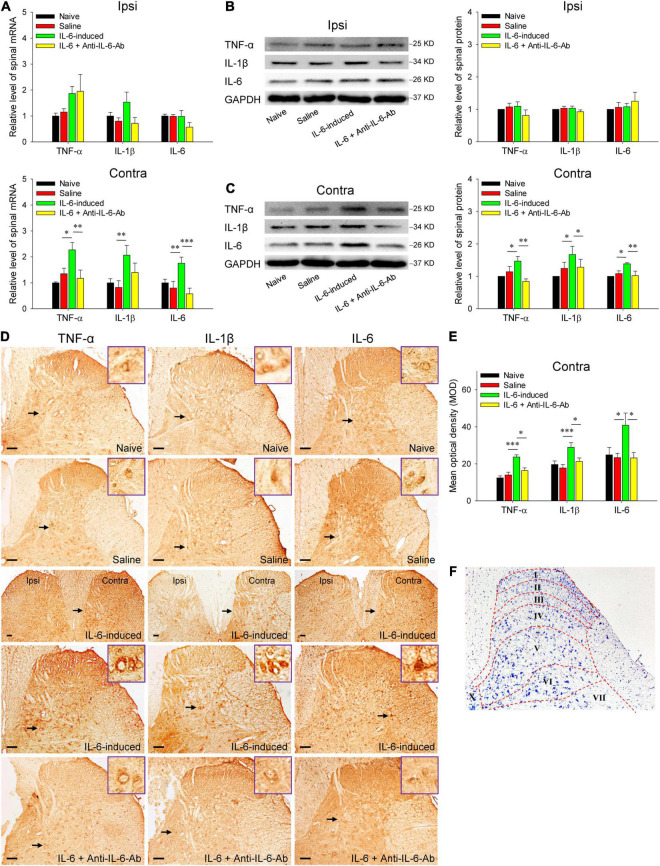
Increased expressions of spinal TNF-α, IL-1β, and IL-6 in rubral IL-6-evoked tactile allodynia rats. **(A)** qRT-PCR showed that intrarubral injection of IL-6 significantly enhanced the mRNA levels of contralateral (but not ipsilateral) spinal TNF-α (*n* = 6 rats/group), IL-1β (*n* = 6–8 rats/group), and IL-6 (*n* = 6 rats/group) compared with normal saline control or IL-6 pre-neutralized with anti-IL-6-Ab control. **(B,C)** Western blotting showed that intrarubral injection of IL-6 significantly increased the protein expressions of contralateral (but not ipsilateral) spinal TNF-α, IL-1β, and IL-6 compared with normal saline or IL-6 pre-neutralized with anti-IL-6-Ab control (*n* = 6 rats/group). **(D,E)** Immunohistochemistry demonstrated that intrarubral injection of IL-6 increased the expressions of spinal TNF-α (*n* = 4–5 rats/group), IL-1β (*n* = 4 rats/group), and IL-6 (*n* = 4 rats/group) in the contralateral spinal dorsal horn (*n* = 4 rats/group). **(F)** Schematic diagram of the Rexed’s laminae of the spinal dorsal horn. *P < 0.05, **P < 0.01, and ***P < 0.001. Contra, contralateral to intrarubral injection of IL-6; Ipsi, ipsilateral to intrarubral injection of IL-6. Scale bars = 100 μm.

To explore the immune molecular mechanisms of rubral IL-6-mediated descending facilitation at the spinal cord level, we first detected the spinal levels of pro-inflammatory cytokines TNF-α, IL-1β, and IL-6 in IL-6-evoked tactile allodynia rats since the disproportion of pro-inflammatory and anti-inflammatory cytokines contributes to the development of chronic pathological pain. The qRT-PCR data delineated that intrarubral injection of IL-6 remarkably increased the mRNA levels of contralateral spinal TNF-α (*t* = 2.698, *P* < 0.05), IL-1β (*t* = 3.228, *P* < 0.01), and IL-6 (*t* = 3.834, *P* < 0.01) rather than that of the ipsilateral, compared with normal saline control. Administration of IL-6 pre-neutralized with anti-IL-6-Ab or normal saline did not significantly alter the mRNA levels of spinal TNF-α, IL-1β, and IL-6 (Figure 3A). Next, there were obvious upregulations in TNF-α (*t* = 2.464, *P* < 0.05), IL-1β (*t* = 2.305, *P* < 0.05), and IL-6 (*t* = 2.718, *P* < 0.05) protein expressions in the contralateral spinal cord in IL-6-evoked tactile allodynia rats when compared with normal saline and IL-6 pre-neutralized with anti-IL-6-Ab control (Figure 3C). The protein expressions of ipsilateral spinal TNF-α, IL-1β, and IL-6 did not change significantly (Figure 3B). Immunohistochemistry confirmed the protein changes and displayed that intrarubral injection of IL-6 obviously increased the expressions of TNF-α (*t* = 4.319, *P* < 0.001), IL-1β (*t* = 3.921, *P* < 0.001), and IL-6 (*t* = 2.484, *P* < 0.05) in the contralateral, but not the ipsilateral, spinal dorsal horn (Figures 3D,E). No significant changes of these cytokines were seen in the spinal ventral horn and white matter region (data were not shown). Referring to the rat spinal atlas in Figure 3F (Paxinos and Watson, 2007), the TNF-α protein changes were mainly distributed in laminae I–II and laminae IV–VI, the IL-1β and IL-6 mainly in laminae IV–VI in the spinal dorsal horn. The above results imply that rubral IL-6 may play descending facilitation and produce an algesic effect through upregulating the expressions of spinal pro-inflammatory cytokines TNF-α, IL-1β, and IL-6.

### Intrarubral Injection of Interleukin-6 Inhibits the Expressions of Spinal Anti-inflammatory Cytokines Transforming Growth Factor-β and Interleukin-10

**FIGURE 4:**
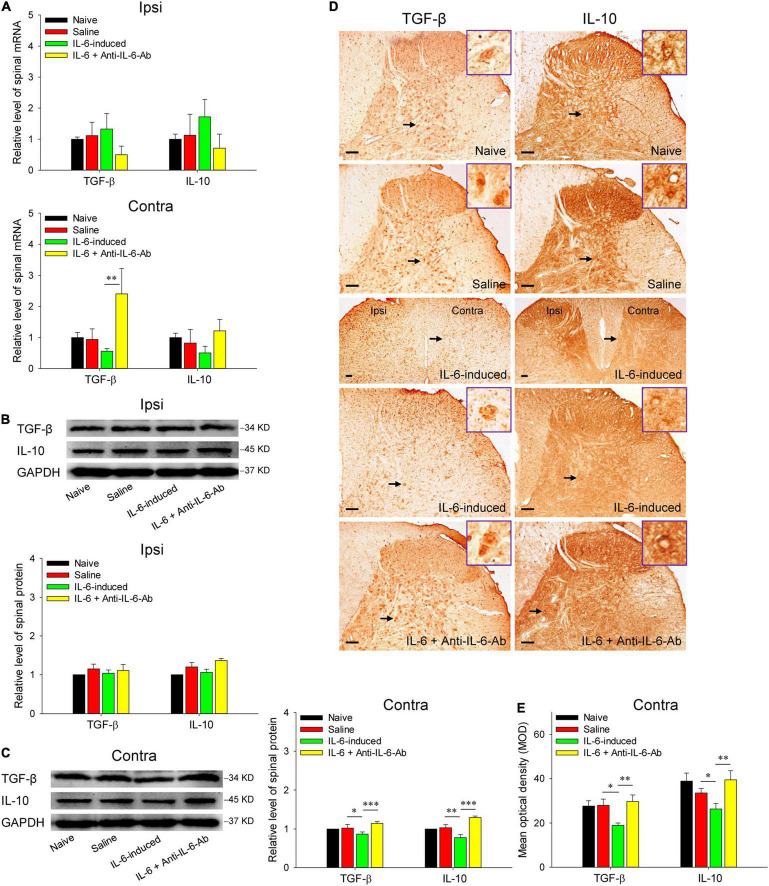
Decreased expressions of spinal TGF-β and IL-10 in rubral IL-6-evoked tactile allodynia rats. **(A)** qRT-PCR showed that intrarubral injection of IL-6 decreased the mRNA levels of contralateral (but not ipsilateral) spinal TGF-β (*n* = 6–8 rats/group) and IL-10 (*n* = 6–8 rats/group) compared with normal saline, although they did not show statistical significance. Administration of IL-6 pre-neutralized with anti-IL-6-Ab even significantly promoted the mRNA expression of spinal TGF-β. **(B,C)** Western blotting showed that intrarubral injection of IL-6 significantly decreased the protein expressions of contralateral (but not ipsilateral) spinal TGF-β and IL-10 compared with normal saline or IL-6 pre-neutralized with anti-IL-6-Ab control (*n* = 6 rats/group). **(D,E)** Immunohistochemistry exhibited that intrarubral injection of IL-6 decreased the expressions of spinal TGF-β (*n* = 4 rats/group) and IL-10 (*n* = 4 rats/group) in the contralateral spinal dorsal horn. *P < 0.05, **P < 0.01, and ***P < 0.001. Contra, contralateral to intrarubral injection of IL-6; Ipsi, ipsilateral to intrarubral injection of IL-6. Scale bars = 100 μm.

Then, we detected the spinal expressions of anti-inflammatory cytokines TGF-β and IL-10 in IL-6-evoked tactile allodynia rats. As shown in Figure 4A, the mRNA levels of anti-inflammatory cytokines TGF-β and IL-10 were downregulated in the contralateral spinal cord post intrarubral injection of IL-6, although the statistics were not significant. However, it is worth noting that administration of IL-6 pre-neutralized with anti-IL-6-Ab promoted the mRNA expression of TGF-β (*t* = 3.211, *P* < 0.01). Further western blotting data indicated intrarubral administration of IL-6 obviously suppressed the protein levels of TGF-β (*t* = 2.278, *P* < 0.05) and IL-10 (*t* = 3.088, *P* < 0.01) in the contralateral but not ipsilateral spinal cord, whereas microinjection of IL-6 pre-neutralized with anti-IL-6-Ab or normal saline did not significantly alter the protein levels of spinal TGF-β and IL-10 (Figures 4B,C). Immunohistochemistry images suggested that both TGF-β (*t* = 2.597, *P* < 0.05) and IL-10 (*t* = 2.111, *P* < 0.05) proteins were mainly decreased in the laminae IV–VI of the contralateral spinal dorsal horn (but not the ipsilateral side) after intrarubral injection of IL-6 (Figures 4D,E). No obvious changes were detected in the spinal ventral horn and white matter region (data were not shown). These results imply that rubral IL-6 may play descending facilitation and produce an algesic effect through downregulating the expressions of spinal anti-inflammatory cytokines TGF-β and IL-10.

### Intrathecal Injection of Neutralizing Antibodies of Tumor Necrosis Factor-α, Interleukin-1β, and Interleukin-6 or Exogenous Transforming Growth Factor-β and Interleukin-10 Attenuated Interleukin-6-Evoked Tactile Allodynia

To explore the actions of these cytokines in rubral IL-6-evoked tactile allodynia, the neutralizing antibodies of TNF-α, IL-1β, and IL-6 or exogenous TGF-β and IL-10 were given intrathecally in advance to observe their effects on rubral IL-6-evoked tactile allodynia. As shown in Figure 2B, pre-intrathecal injection of 2.0 μg anti-TNF-α-Ab remarkably raised the PWT of rats and alleviated rubral IL-6-evoked tactile allodynia (*F* = 23.059, *P* < 0.01), the anti-allodynic effect initiated at 15 min post injection and sustained for 45 min. Similarly, pre-injection of 1.0 μg anti-IL-1β-Ab (*F* = 12.906, *P* < 0.05) or 1.0 μg anti-IL-6-Ab (*F* = 6.312, *P* < 0.05) into the spinal cord also alleviated rubral IL-6-evoked tactile allodynia, the anti-allodynic effect of both started at 45 min post injection and sustained for 30 min (Figures 2C,D). There are no significant changes in the PWT of rats after intrathecal injection of anti-TNF-α-Ab, anti-IL-1β-Ab, or anti-IL-6-Ab alone. Footprint data showed that intrathecal administration of antibodies against TNF-α, IL-1β, or IL-6 had no impact on the locomotion of rats (Figure 2G). These results confirm that rubral IL-6 mediates descending facilitation by upregulating the expressions of spinal TNF-α, IL-1β, and IL-6.

To further probe the function of spinal TGF-β and IL-10, recombinant TGF-β or IL-10 was intrathecally injected 5 min before intrarubral injection of IL-6. Results revealed that intrathecal injection of 10 ng TGF-β could elevate the PWT and alleviate the tactile allodynia induced by rubral IL-6 (*F* = 61.403, *P* < 0.001). The analgesic effect started at 15 min and sustained for 60 min (Figure 2E). Intrathecal injection of 300 ng IL-10 before intrarubral injection of IL-6 also raised the PWT, and the effect acted from 15 min and sustained for 45 min (*F* = 26.262, *P* < 0.01) (Figure 2F). There are no significant changes in the PWT of rats after intrathecal injection of TGF-β or IL-10 alone. Footprint data showed that intrathecal injection of recombinant rat TGF-β or IL-10 did not influence motor behavior (Figure 2G). These findings confirm that rubral IL-6 mediates descending facilitation through downregulating the expressions of spinal TGF-β and IL-10.

### Red Nucleus Interleukin-6 Regulates Spinal Pro-inflammatory and Anti-inflammatory Cytokines by Janus Kinase 2/Signal Transducer and Activator of Transcription 3 and/or Extracellular Signal-Regulated Kinase Pathways

**FIGURE 5:**
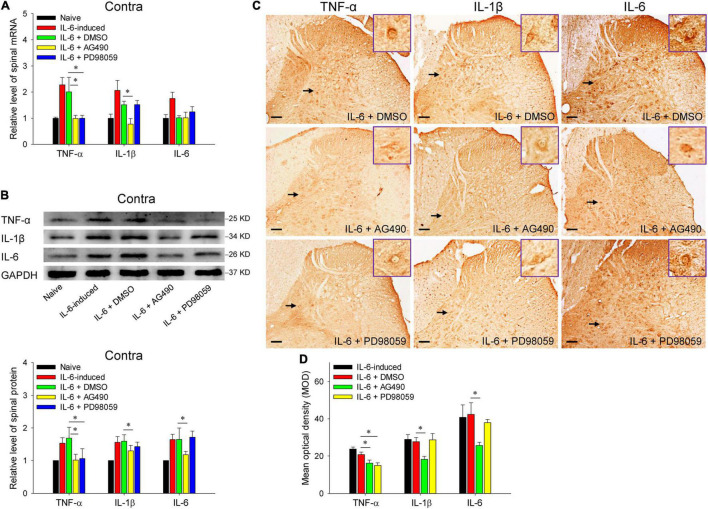
Red nucleus IL-6 induced the contralateral spinal TNF-α, IL-1β, and IL-6 by activating JAK2/STAT3 and/or ERK pathways. **(A)** qRT-PCR displayed that intrarubral pre-injection of JAK2/STAT3 inhibitor AG490 significantly downregulated the mRNA expressions of contralateral spinal TNF-α (*n* = 6–8 rats/group) and IL-1β (*n* = 6–8 rats/group), but ERK inhibitor PD98059 only notably lowered the mRNA of spinal TNF-α compared with DMSO control. **(B)** Western blotting showed that intrarubral pre-injection of JAK2/STAT3 inhibitor AG490 significantly downregulated the protein levels of contralateral spinal TNF-α, IL-1β, and IL-6, ERK inhibitor PD98059 only significantly reduced the protein levels of spinal TNF-α compared with DMSO control (*n* = 6 rats/group). **(C,D)** Immunohistochemistry demonstrated that intrarubral pre-injection of JAK2/STAT3 inhibitor AG490 downregulated the protein levels of spinal TNF-α (*n* = 4–5 rats/group), IL-1β (*n* = 4 rats/group), and IL-6 (*n* = 4 rats/group), and ERK inhibitor PD98059 reduced the protein levels of spinal TNF-α in the contralateral spinal dorsal horn. *P < 0.05. Contra, contralateral to intrarubral injection of IL-6. Scale bars = 100 μm.

**FIGURE 6:**
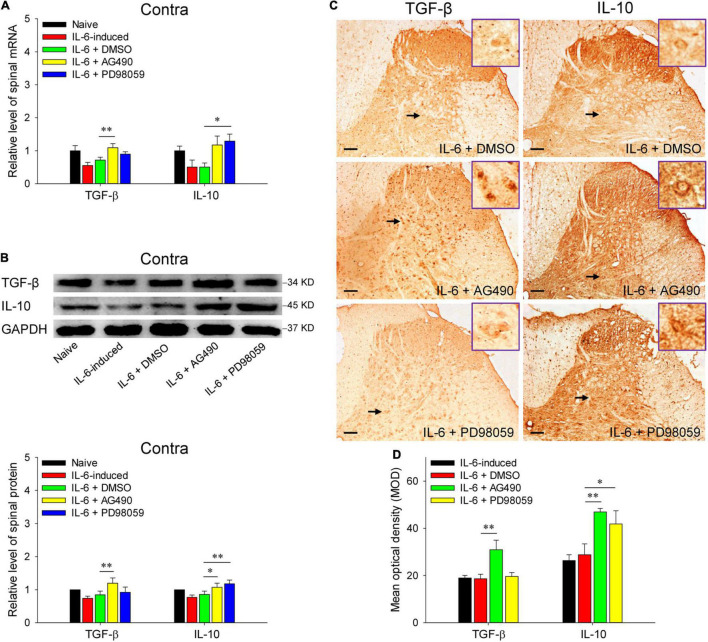
Red nucleus IL-6 inhibited the contralateral spinal TGF-β and IL-10 by activating JAK2/STAT3 and/or ERK pathways. **(A)** qRT-PCR showed that intrarubral pre-injection of JAK2/STAT3 inhibitor AG490 significantly promoted the mRNA expression of contralateral spinal TGF-β (*n* = 6–8 rats/group), while ERK inhibitor PD98059 significantly enhanced the mRNA expression of IL-10 (*n* = 6–8 rats/group) compared with DMSO control. **(B)** Western blotting showed that intrarubral pre-injection of JAK2/STAT3 inhibitor AG490 promoted the protein expressions of the contralateral spinal TGF-β and IL-10, but ERK inhibitor PD98059 only enhanced the protein expression of IL-10 compared with DMSO control (*n* = 6 rats/group). **(C,D)** Immunohistochemistry exhibited that intrarubral pre-injection of JAK2/STAT3 inhibitor AG490 promoted the protein expressions of spinal TGF-β (*n* = 4 rats/group) and IL-10 (*n* = 4 rats/group), and ERK inhibitor PD98059 enhanced the protein expression of IL-10 in the contralateral spinal dorsal horn. *P < 0.05 and **P < 0.01. Contra, contralateral to intrarubral injection of IL-6. Scale bars = 100 μm.

Reminiscent of our previous results, rubral IL-6 activates JAK2/STAT3 and ERK pathways in regulating the maintenance of chronic pain (Ding et al., 2018). Here, we further investigated the pathways by which rubral IL-6 regulates spinal pro-inflammatory and anti-inflammatory cytokines. Our results proved that pre-injection of JAK2/STAT3 inhibitor AG490 significantly downregulated the mRNA expressions of spinal TNF-α (*t* = 2.671, *P* < 0.05) and IL-1β (*t* = 2.407, *P* < 0.05) (Figure 5A), while promoted the mRNA expression of spinal TGF-β (*t* = 3.359, *P* < 0.01) (Figure 6A). Although pre-injection of AG490 also could decrease the mRNA expression of spinal IL-6 and increase the mRNA expression of spinal IL-10, there were no statistic significant (Figures 5A, 6A). However, both western blotting and immunohistochemistry results indicated that intrarubral pre-administration of AG490 could remarkably decrease the protein levels of spinal TNF-α (*t* = 2.745, *P* < 0.05; *t* = 2.072, *P* < 0.05), IL-1β (*t* = 2.758, *P* < 0.05; *t* = 2.336, *P* < 0.05), and IL-6 (*t* = 2.172, *P* < 0.05; *t* = 2.678, *P* < 0.05) (Figures 5B–D), and enhance the protein expressions of spinal TGF-β (*t* = 2.928, *P* < 0.01; *t* = 3.872, *P* < 0.01) and IL-10 (*t* = 2.338, *P* < 0.05; *t* = 3.122, *P* < 0.01) in IL-6-evoked tactile allodynia rats (Figures 6B–D). Unlike the AG490, intrarubral pre-injection of ERK inhibitor PD98059 only significantly reduced the mRNA and protein of spinal TNF-α (*t* = 2.699, *P* < 0.05; *t* = 2.553, *P* < 0.05; *t* = 2.540, *P* < 0.05) and increased the mRNA and protein of IL-10 (*t* = 2.103, *P* < 0.05; *t* = 3.453, *P* < 0.01; *t* = 2.238, *P* < 0.05) in IL-6-evoked tactile allodynia rats (Figures 5A–D, 6A–D). Above evidence reveals that rubral IL-6 activates JAK2/STAT3 and/or ERK pathways to induce spinal pro-inflammatory cytokines, while inhibit spinal anti-inflammatory cytokines to evoke tactile allodynia.

## Discussion

It is well known that IL-6 is a pleiotropic inflammatory cytokine and has a widespread distribution in the central and peripheral nervous systems. Recently, IL-6 has been related to the modulation of chronic pathological pain, and an upregulation of IL-6 is discovered in the spinal cord and dorsal ganglion at pain state (Zhou et al., 2016). We have previously confirmed that IL-6 is expressed in the RN of naive rats and enhanced in the RN at 3 weeks post peripheral nerve injury. Blocking the effect of rubral IL-6 relieves pathological pain induced by nerve injury, while intrarubral administration of exogenous IL-6 can dose-dependently evoke obvious tactile allodynia in naive rats, indicating that IL-6 yields a facilitatory effect in the maintenance of neuropathic pain (Ding et al., 2016; Yang et al., 2020). In this study, we have further explored the immune molecular mechanisms of rubral IL-6-mediated descending facilitation at the spinal cord level by using rubral IL-6-evoked tactile allodynia rats. A series of studies have indicated that IL-6 and microglia, one of the main IL-6 producing cells, have gender differences in modulating the activity, neuroinflammation, and pain (Miller et al., 2010; Aniszewska et al., 2014; Halievski et al., 2020; Mun et al., 2020), therefore only male rats are adopted in this study. In IL-6-evoked tactile allodynia rats, a transient but obvious tactile allodynia is evoked in the contralateral but not ipsilateral hindpaw following intrarubral injection of IL-6 in naive rats. Since the tactile allodynia evoked by rubral IL-6 avoids the interference of other factors, it is fit for studying the algesic mechanisms of rubral IL-6. Additionally, intrarubral administration of IL-6 only evokes obvious tactile allodynia in the contralateral hindpaw, which further supports the fact that the RN mainly controls the contralateral distal limb (Liang et al., 2012).

Pro-inflammatory cytokines TNF-α, IL-1β, and IL-6 and anti-inflammatory cytokines TGF-β and IL-10 are identified contributory factors of nociception and chronic pain. Consistent with other literature reporting changes of spinal pro-inflammatory cytokines in neuropathic pain (Liu et al., 2017; Ho et al., 2020; Kwok et al., 2020), we have observed that intrarubral injection of IL-6 induces the increased mRNA and protein expressions of TNF-α, IL-1β, and IL-6 in the contralateral spinal cord. Blocking spinal TNF-α, IL-1β, or IL-6 with antibodies all obviously attenuates IL-6-evoked tactile allodynia, of which anti-TNF-α-Ab has a longer duration of effectiveness than antibodies against IL-1β and IL-6. Based on other studies (Shamash et al., 2002; Zhang et al., 2011; König et al., 2016), spinal TNF-α may play a more vital action in IL-6-evoked tactile allodynia. Conversely, intrarubral injection of IL-6 decreases the mRNA and protein expressions of TGF-β and IL-10 in the contralateral spinal cord, although no statistical significance exists in mRNA changes of TGF-β and IL-10. The discrepancies may result from the temporal and spatial gaps between mRNA transcription and protein translation (Buccitelli and Selbach, 2020). Additionally, a significant increase in mRNA expression of TGF-β has been detected post injection of IL-6 pre-neutralized with anti-IL-6-Ab, which is possible that endogenous IL-6 suppresses TGF-β under physiological conditions, and excess antibodies block the effect of endogenous IL-6, causing an increase in TGF-β mRNA. Intrathecal administration of exogenous TGF-β or IL-10 proteins generates an analgesic effect on rubral IL-6-evoked tactile allodynia rats, which is in line with other reports that the IL-10 and TGF-β function as anti-hypersensitive factors in neuropathic pain (Chen et al., 2013; Wu et al., 2018). Histochemical images show except for TNF-α, which is also altered in laminae I–II, the protein changes of TNF-α, IL-1β, IL-6, TGF-β, and IL-10 are mainly in the laminae IV–VI of dorsal horn, which corresponds to the projection region of the RST. Laminae V–VI is usually identified as the main area receiving afferents from innocuous stimuli and may be involved in integrated motor and sensory regulation (Todd, 2013; Peirs and Seal, 2016; MacKinnon, 2018). We speculate that intrarubral injection of IL-6 causes the sensitization of laminae V–VI, which amplifies the received innocuous signals and further transmits them to the supraspinal region leading to allodynia. In a word, rubral IL-6 evokes tactile allodynia by promoting the expressions of spinal TNF-α, IL-1β, and IL-6 while suppressing the expressions of spinal IL-10 and TGF-β. Although our study has established a link between the rubral and spinal cytokines in the regulation of pain, the mechanisms of rubral IL-6 modulating spinal inflammatory milieu to facilitate pain remains unclear. Our previous study has demonstrated that IL-6 and IL-6 receptor (IL-6R) in the RN of normal rats are mainly distributed in neurons and oligodendrocytes (Ding et al., 2016). Thus, it implies that rubral IL-6 may directly activate the neurons of RST through binding to neuronal IL-6R, and then further activate spinal neurons and also glial cells, leading to the expression changes of pro-inflammatory and anti-inflammatory cytokines. Further studies are needed to establish precise mechanisms.

Interleukin-6 signaling is known to function by activating the JAK/STAT3 as well as MAPK/ERK system in various pain conditions (Dominguez et al., 2008; Manjavachi et al., 2010; Yan et al., 2012; Fang et al., 2015). Based on the role of JAK2/STAT3 and ERK in rubral IL-6-mediated maintenance of neuropathic pain (Ding et al., 2016, 2018; Yang et al., 2020), in this study, we find that antagonizing JAK2/STAT3 not only inhibits rubral IL-6 induced expressions of spinal TNF-α, IL-1β, and IL-6 but also promotes the expressions of spinal TGF-β and IL-10. Blocking the ERK pathway only restrains the effect of rubral IL-6 on spinal TNF-α and IL-10. Although the mRNA expressions changes of spinal IL-6 and IL-10 are not statistically significant, the trend is consistent with that of proteins. The above results display that rubral IL-6 induces spinal pro-inflammatory cytokines and inhibits spinal anti-inflammatory cytokines *via* activating rubral JAK2/STAT3 and/or ERK pathways. However, it is unclear whether or not spinal JAK2/STAT3 and ERK are also involved in rubral IL-6-evoked tactile allodynia, although some previous studies have shown that spinal JAK/STAT3 and ERK pathways play a vital role in different pain conditions by regulating the expressions of pro-inflammatory and anti-inflammatory cytokines (Ji et al., 2014; Wu et al., 2018).

In sum, we have investigated the immune molecular mechanisms of rubral IL-6-mediated pain regulation at the spinal level. Rubral IL-6 exerts an algesic effect by stimulating spinal pro-inflammatory cytokines TNF-α, IL-1β, and IL-6 and suppressing spinal TGF-β and IL-10. Rubral IL-6 modulates spinal pro-inflammatory and anti-inflammatory cytokines *via* JAK2/STAT3 and/or ERK pathways. However, the molecular mechanisms of rubral IL-6-mediated pain regulation at the peripheral nervous system (including DRG and peripheral nerves) are still unclear, and further investigation is needed. Our study proves that rubral IL-6 plays a descending facilitatory effect in the modulation of pain, targeting IL-6 itself or its downstream signaling pathways and molecules may be a novel pain therapeutic.

## Data Availability Statement

The raw data supporting the conclusions of this article will be made available by the authors, without undue reservation.

## Ethics Statement

The animal study was reviewed and approved by the Biomedical Ethics Committee of Xi’an Jiaotong University.

## Author Contributions

J-YW and X-YZ designed the study. Q-QY, H-NL, Y-TX, XT, FF, JY, Y-LX, JG, and X-QL carried out the experiments. J-YW, Q-QY, and H-NL performed the statistical analysis and prepared the manuscript. All authors approved the submitted version.

## Conflict of Interest

The authors declare that the research was conducted in the absence of any commercial or financial relationships that could be construed as a potential conflict of interest.

## Publisher’s Note

All claims expressed in this article are solely those of the authors and do not necessarily represent those of their affiliated organizations, or those of the publisher, the editors and the reviewers. Any product that may be evaluated in this article, or claim that may be made by its manufacturer, is not guaranteed or endorsed by the publisher.
